# Sampling variability between two mid-turbinate swabs of the same patient has implications for influenza viral load monitoring

**DOI:** 10.1186/s12985-014-0233-9

**Published:** 2014-12-24

**Authors:** Liesbeth Van Wesenbeeck, Hanne Meeuws, David D’Haese, Gabriela Ispas, Lieselot Houspie, Marc Van Ranst, Lieven J Stuyver

**Affiliations:** Janssen Infectious Diseases - Diagnostics BVBA, Turnhoutseweg 30, B2340 Beerse, Belgium; Laboratory of Clinical Virology, Rega Institute for Medical Research, KU Leuven, Minderbroedersstraat 10, B3000 Leuven, Belgium

**Keywords:** Influenza viral load, Nasal swabs, Sampling

## Abstract

**Background:**

With the clinical development of several antiviral intervention strategies for influenza, it becomes crucial to explore viral load shedding in the nasal cavity as a biomarker for treatment success, but also to explore sampling strategies for sensible and reliable virus collection.

**Findings:**

In this study, 244 patients suffering from Influenza like Illness and/or acute respiratory tract infection were enrolled. Sampling was done using mid-turbinate flocked swabs and two swabs per patient were collected (one swab per nostril). The influenza A viral loads of two mid-turbinate flocked swabs (one for each nostril) per patient were compared and we have also assessed whether normalization for human cellular DNA in the swabs could be useful. The Influenza mid-turbinate nasal swab testing resulted in considerable sampling variability that could not be normalized against co-isolated human cellular DNA.

**Conclusions:**

Influenza viral load monitoring in nasal swabs could be very valuable as virological endpoints in clinical trials to monitor treatment efficacy, in analogy to HIV, HBV & HCV viral load monitoring. However, the differences between left and right nostrils, as observed in this study, highlight the importance of proper sampling and the need for standardized sampling procedures.

## Findings

Respiratory tract infections (RTI) have an enormous social economic impact, with high incidence of hospitalization and high costs [1,2]. Because of similar clinical symptoms and simultaneous circulation of several different viruses, the etiology is often unknown. Timely detection and discrimination of the infecting pathogens is crucial to optimize treatment and care, to prevent unnecessary antibiotic use, and to prevent secondary spread of infection. Adequate specimen collection is the first crucial step for the correct diagnosis of influenza and other respiratory infections. Correction for the dilution in nasopharyngeal aspirates might improve the detection of respiratory infections [3,4]. Many studies have described that mid-turbinate flocked swabs are less invasive than other specimen collection types (nasopharyngeal swabs, aspirates & washes), have a good sensitivity to detect respiratory viruses and are therefore a good alternative for specimen collection [5-11]. Moreover, these mid-turbinate swabs have the possibility for self-collection (either adult patients or parents of children at home) [5-11]. To our knowledge, these studies have compared the different specimen types and/or specimen collection by a health care worker and self-collection, but none of them have compared the variability of respiratory virus detection between the two nostrils of the same patient at the same time point. In HIV, HCV, and HBV, viral load monitoring is a key diagnostic parameter to measure treatment success [12-14]. With the clinical development of several antiviral intervention strategies for influenza, it becomes crucial to explore viral load shedding in the nasal cavity as a biomarker for treatment success, but also to explore sampling strategies for sensible and reliable virus collection. In this study, the influenza A viral loads of two mid-turbinate flocked swabs (one for each nostril) per patient were compared. We have also assessed whether normalization for human cellular DNA in the swabs could be useful.

A total of 244 patients suffering from Influenza Like Illness (ILI) and/or acute respiratory tract infection (RTI), presenting for medical support at a primary care physicians’ office in Belgium, were enrolled between February-March 2012 and January-March 2013, as previously described [15]. The study was approved by the Committee on Medical Ethics of the University Hospital of Leuven in Belgium and written consent from all participants was obtained. Only patients symptomatic for fewer than 3 days were included in the study. Sampling was done using mid-turbinate flocked swabs (Copan, Italy) and two swabs per patient were collected (one swab per nostril; info on the order of the swab collections (left/right nostril) is not available). One swab was re-suspended in 1 ml Universal transport medium (UTM) (swab A) and the other swab in 3 ml UTM (swab B) to define if the swab collection in a smaller volume results in a higher viral load. Swabs were kept refrigerated (4°C) until transport to central lab where the samples were stored at -80°C. Viral RNA was isolated using the Easymag system (Biomerieux) and Influenza viral load was determined in each swab using qRT-PCR, which was performed according to our in-house protocol for Influenza A (InfA) (targeting the Matrix gene) (based on the CDC protocol [16]) with a panel of oligonucleotide primers and dual-labeled hydrolysis (TaqMan®) probes, as previously described [15]. Each sample was tested in duplicate. The InfA qRT-PCR is characterized by a low inter- and intra-variability (average %CV < 2.5%) (data not shown). All Ct values were corrected for the loss of RNA during extraction by use of the internal extraction control (IEC) [17]. A standard RNA dilution series (8 dilutions; External quantification control (EQC)) was tested in duplicate in each qRT-PCR experiment [15]. The viral loads of the processed samples were calculated as log_10_ copies/ml through back calculation on the EQC standard curve, which has the following characteristics y = -3.03x + 40.17 with r^2^ = 0.99 and a linear range from 4.3 to 10.3 log_10_ copies/ml (lower and upper limit of quantification (LLOQ and ULOQ)). All samples with an InfA viral load below 4.3 log_10_ copies/ml were defined as ‘below LLOQ’. To detect the amount of human cells in the mid-turbinate swabs, qPCR (on DNA level) was performed with primers/probe located on the human RNaseP gene, as proposed by CDC [16]. Each sample was tested in duplicate. Amplification and detection were performed on Lightcycler® 480 instrument (Roche Applied Science). The 25 μl reaction mixture of the qPCR was comprised of 5 μl of nucleic acid, 12.5 μl of 2x PCR master mix, 0.5 μl of Platinum® *Taq* Mix (Life Technologies), 0.5 μl of forward and reverse primers (40 μM) and 0.5 μl of labeled probe (10 μM), and 5.5 μl water. The thermal cycling conditions were as follows: 95°C for 2 min, followed by 45 cycles of PCR amplification (95°C for 15 s and 55°C for 30 s). A 1/10 dilution series of human control genomic DNA (Applied Biosystems) was tested in duplicate on each real-time PCR plate and has the following characteristics: y = -3.33x + 40.04 with r^2^ = 0.99.

**Figure 1:**
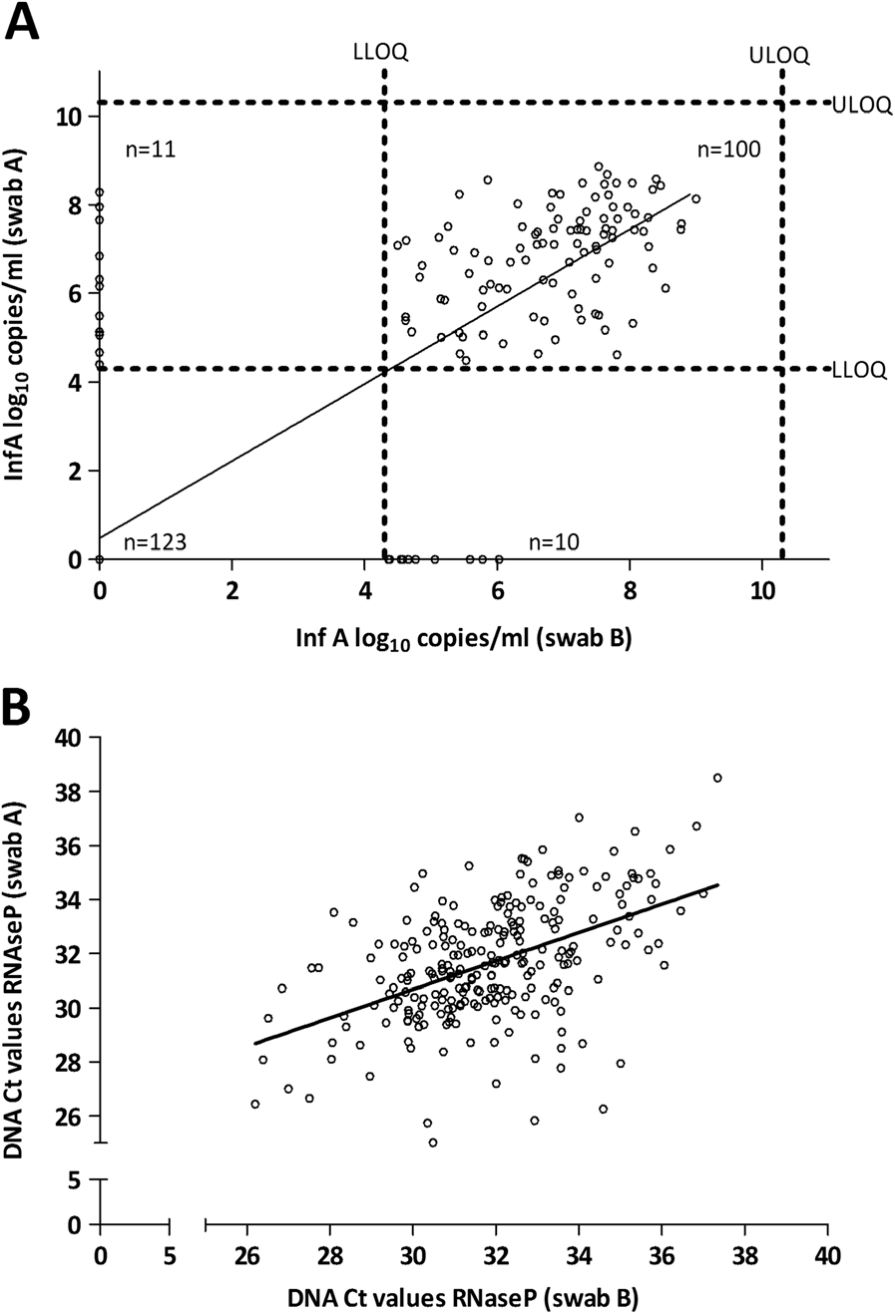
**Variability plot of influenza A viral load or RnaseP DNA as observed for two mid-turbinate swabs taken from the same patient (n = 244). A:** Influenza A viral load (r2=0.733): One hundred samples have an InfA VL > LLOQ for both swabs (r2 = 0.183). Dotted lines represent upper limit of quantification (ULOQ) = 10.3 log10 copies/ml & lower limit of quantification (LLOQ) = 4.3 log10 copies/ml. Viral load values below LLOQ are plotted in the respective axes or on the origin. **B:** RnaseP DNA (r2 = 0.242).

**Figure 2:**
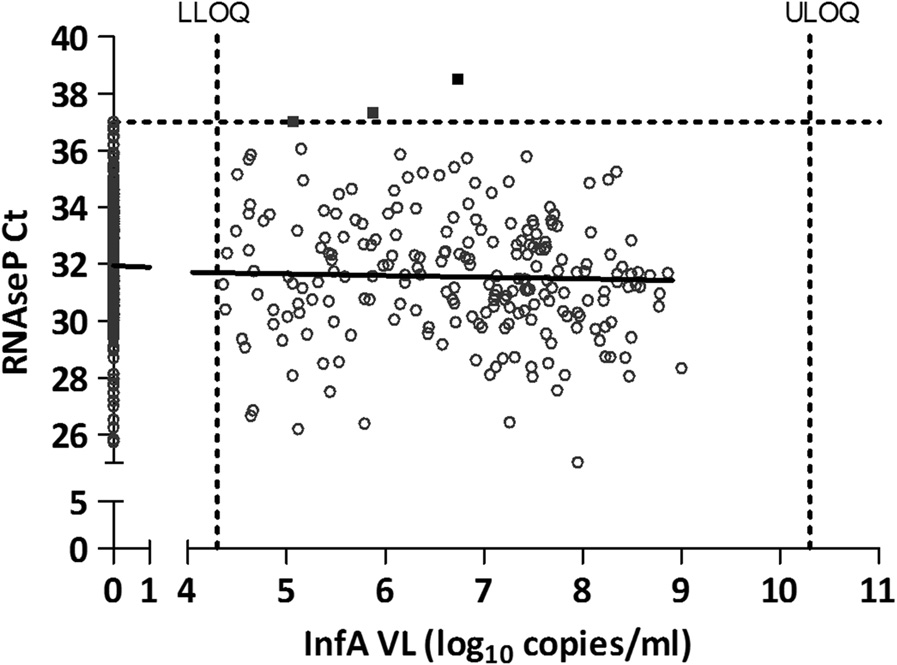
**Plot of the RNaseP Ct values versus InfA log**_**10**_ **copies/ml (n = 488; two swabs/patient) (r** ^**2**^ **= 0,009 and slope = -0,06).** Dotted lines represent LLOQ & ULOQ of the InfA viral load assay.

**Figure 3:**
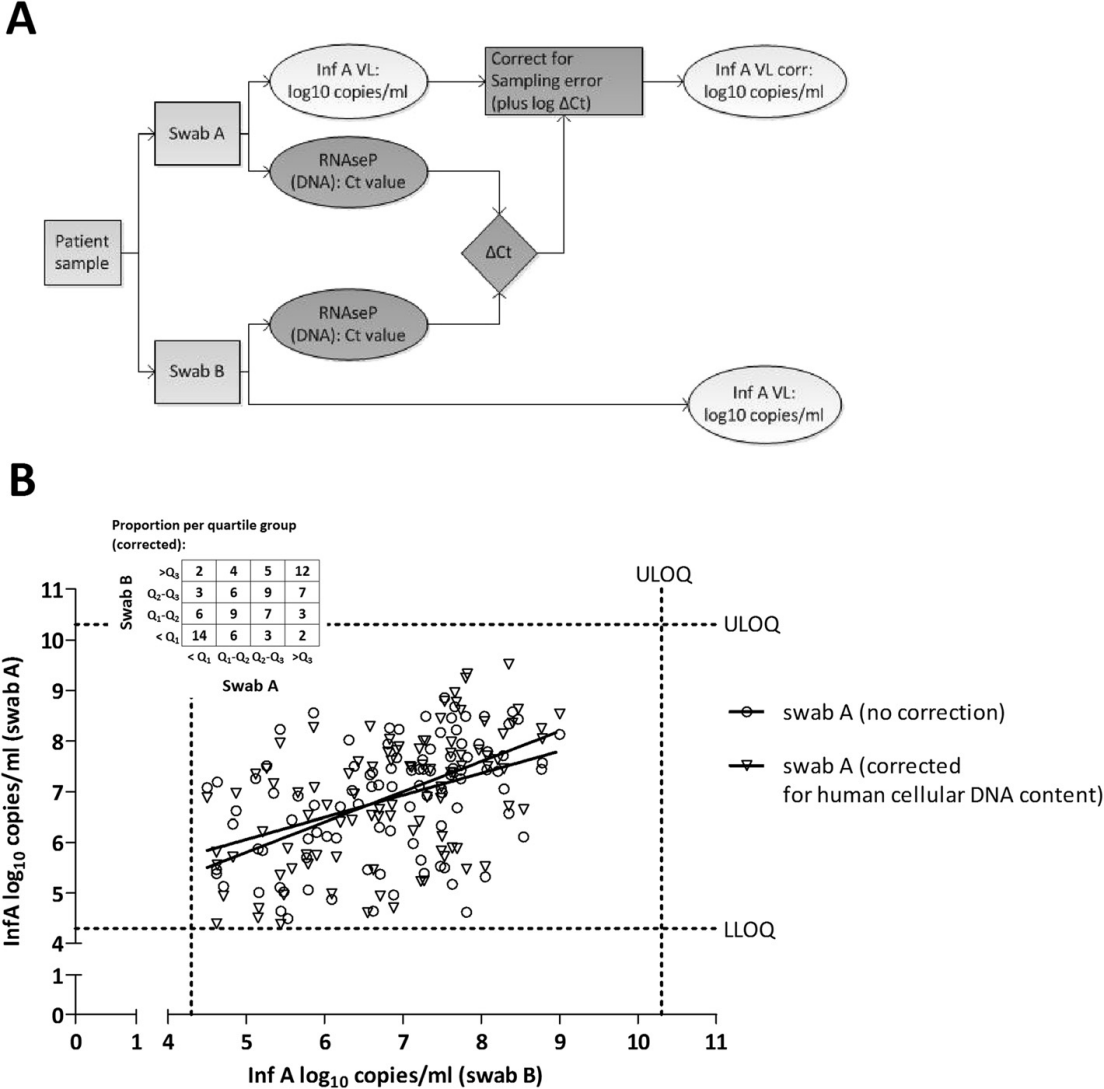
**Correction of the influenza A viral load for amount of human cells in the swabs. A:** Process flow. **B:** Comparison of the influenza A viral load of the swabs from both nostrils before and after correction for human cell content (n=100). Values < LLOQ are omitted. The Swab A and B InfA viral load (corrected for human cellular DNA) were classified into 4 groups delimited by the first, second and third quartile and the relative frequency for each group were tabulated. (VL=viral load).

Two hundred forty four (244) patients were enrolled in this study, resulting in a total of 488 swabs. Collection of the swab in a smaller volume (Swab A) resulted in a 6.3 times higher InfA viral load compared to swab B. For all subsequent analyses, the difference in volume was mathematically corrected by adding 1.5 Ct to the obtained Ct values of swab A. For 100 patients (41.0%), an InfA viral load between 4.3 & 10.3 log_10_ copies/ml was detected on both mid-turbinate swabs. One hundred and twenty three patients (50.4%) had two swabs with an InfA viral load below LLOQ. For 21 patients (8.6%) only one swab was positive for InfA (the other swab was < LLOQ for infA) (Figure 1A). The average InfA viral load of swabs A & B of the 100 influenza A positive patients was almost identical (6.86 log_10_ copies/ml for swab A and 6.84 log_10_ copies/ml for swab B). In this subset of samples (n = 100), a high variability of the infA viral load between the left and right nostril was observed (r^2^ = 0.183). Eleven samples (out of the 100 concordant positive samples) had an InfA viral load difference of more than 2 log_10_ copies/ml (Figure 1A). A wide range of human RNaseP DNA content was found in the 488 samples (range: 25.02 Ct - 38.51 Ct with an average of 31.76 Ct) (Figure 1B). A poor correlation between the RnaseP Ct value of two swabs of the same patient was found (r^2^ = 0.240). A very low amount of human DNA was found in only 3 samples (Ct value of RNaseP >37), illustrating that the majority of the samples contained a detectable amount of human DNA. As shown in Figure 2, the RNaseP DNA content is independent of the influenza A viral load. Indeed, InfA viral load between 5.06 & 6.73 log_10_ copies/ml was found in the 3 samples with a low amount of human DNA (Ct value of RNaseP >37). Figure 3A describes the process flow, which is performed on the 100 concordant infA positive samples, to correct the InfA viral load for the difference in cell content between the two swabs of a patient. The difference between RNaseP Ct values of the two swabs of the same patient was calculated. The log conversion of this ∆Ct value of RNAseP was added to the InfA viral load of swab A. This corrected InfA viral load of swab A can then be plotted against the viral load of swab B. As shown in Figure 3B, only a slight improvement of the correlation between both swabs of the same patient can be observed (not corrected swab A (r^2^ = 0.183; mean of difference = 0.02 ± 1.21) versus swab A corrected for sampling (r^2^ = 0.286; mean of difference = 0.07 ± 1.15)). To address the issue of the correlation varying across the range of VL values, the Swab A and B InfA viral load (corrected for human cellular DNA) were classified into 4 groups delimited by the first, second and third quartile and the relative frequency for each group were tabulated as an insert to Figure 3B.

Flu surveillance studies focus on identifying the circulating influenza viruses and their main characteristics, independent of the viral quantity. Influenza viral load monitoring (on different timepoints) in nasal swabs could be very valuable as virological endpoints in clinical trials to monitor treatment efficacy, in analogy to HIV, HBV & HCV viral load monitoring. The differences in InfA viral load between the left and right nostrils, as observed in this study, could be related to variability of the qRT-PCR, biological differences between the nostrils, sampling bias or any combination of these. The qRT-PCR is characterized by a low intra- and inter variability (CV < 2.5%). A right- or left-handed person may obtain the swab differently from the right/left nostril or the order of taking the swabs could also introduce variability. Unfortunately, that info was not collected in this study but this should be included in further studies. The biological differences could include anatomic nose/septum abnormalities, variability in virus shedding or clogging of the nostrils. The differences in Inf A viral load between left and right nostrils, as observed in this study, highlight the importance of proper sampling and the need for standardized sampling procedures. Several options for sampling can be proposed: one swab per patient (which is now the case for most studies), two swabs per patient (one for each nostril), two swabs per patient (one for each nostril) with collection in the same vial, the use of one swab for sampling of both nostrils. Further studies are definitely needed to further explore the use of mid-turbinate swabs, the variability between the nostrils of a patient and to propose one standard procedure for specimen collection which would improve the adequate detection of influenza/respiratory viruses and in the end will contribute to adequately monitor treatment efficacy in clinical trials.

## References

[1] Joseph C, Togawa Y, Shindo N (2013). Bacterial and viral infections associated with influenza. Influenza Other Respi Viruses.

[2] Fineberg HV (2014). Pandemic preparedness and response–lessons from the H1N1 influenza of 2009. N Engl J Med.

[3] Heikkinen T, Shenoy M, Goldblum RM, Chonmaitree T (1999). Free secretory component as a standardization protein for nasopharyngeal specimens from children with upper respiratory tract infection. Acta Paediatr.

[4] Heikkinen T, Shenoy M, Goldblum RM, Chonmaitree T (1999). Quantification of cytokines and inflammatory mediators in samples of nasopharyngeal secretions with unknown dilution. Pediatr Res.

[5] Larios OE, Coleman BL, Drews SJ, Mazzulli T, Borgundvaag B, Green K, McGeer AJ, Group ST-FS (2011). Self-collected mid-turbinate swabs for the detection of respiratory viruses in adults with acute respiratory illnesses. PLoS One.

[6] Esposito S, Molteni CG, Daleno C, Valzano A, Tagliabue C, Galeone C, Milani G, Fossali E, Marchisio P, Principi N (2010). Collection by trained pediatricians or parents of mid-turbinate nasal flocked swabs for the detection of influenza viruses in childhood. Virol J.

[7] Abu-Diab A, Azzeh M, Ghneim R, Ghneim R, Zoughbi M, Turkuman S, Rishmawi N, Issa AE, Siriani I, Dauodi R, Kattan R, Hindiyeh MY (2008). Comparison between pernasal flocked swabs and nasopharyngeal aspirates for detection of common respiratory viruses in samples from children. J Clin Microbiol.

[8] Heikkinen T, Marttila J, Salmi AA, Ruuskanen O (2002). Nasal swab versus nasopharyngeal aspirate for isolation of respiratory viruses. J Clin Microbiol.

[9] Heikkinen T, Salmi AA, Ruuskanen O (2001). Comparative study of nasopharyngeal aspirate and nasal swab specimens for detection of influenza. BMJ.

[10] Smieja M, Castriciano S, Carruthers S, So G, Chong S, Luinstra K, Mahony JB, Petrich A, Chernesky M, Savarese M, Triva D (2010). Development and evaluation of a flocked nasal midturbinate swab for self-collection in respiratory virus infection diagnostic testing. J Clin Microbiol.

[11] Daley P, Castriciano S, Chernesky M, Smieja M (2006). Comparison of flocked and rayon swabs for collection of respiratory epithelial cells from uninfected volunteers and symptomatic patients. J Clin Microbiol.

[12] Stephan C, Hill A, Sawyer W, van Delft Y, Moecklinghoff C (2013). Impact of baseline HIV-1 RNA levels on initial highly active antiretroviral therapy outcome: a meta-analysis of 12,370 patients in 21 clinical trials*. HIV Med.

[13] Perry CM (2012). Telaprevir: a review of its use in the management of genotype 1 chronic hepatitis C. Drugs.

[14] Garnock-Jones KP (2012). Boceprevir: a review of its use in the management of chronic hepatitis C genotype 1 infection. Drugs.

[15] Van Wesenbeeck L, Meeuws H, Van Immerseel A, Ispas G, Schmidt K, Houspie L, Van Ranst M, Stuyver L (2013). Comparison of the FilmArray RP, Verigene RV+, and Prodesse ProFLU+/FAST+ multiplex platforms for detection of influenza viruses in clinical samples from the 2011-2012 influenza season in Belgium. J Clin Microbiol.

[16] WHO (2009). CDC Protocol of Real-Time RT-PCR for Swine Influenza A(H1N1). Book CDC Protocol of Real-Time RT-PCR for Swine Influenza A(H1N1).

[17] Nauwelaers D, Vijgen L, Atkinson C, Todd A, Geretti AM, Van Ranst M, Stuyver L (2009). Development of a real-time multiplex RSV detection assay for difficult respiratory samples, using ultrasone waves and MNAzyme technology. J Clin Virol.

